# Environmental Cardiology: Getting to the Heart of the Matter

**DOI:** 10.1289/ehp.112-a880

**Published:** 2004-11

**Authors:** Bob Weinhold

Cardiovascular disease (CVD) is the leading killer in many developed countries, and is soon expected to be the leading killer in all countries. A number of factors have raised CVD to this unsavory stature, among them lack of exercise, poor diet, and smoking. But evidence has slowly been building to indicate that exposures to chemicals and other environmental substances also can have a profound impact on heart health. The link between environmental agents and CVD was once considered tenuous by much of the medical and scientific establishment. But after watching the evidence accumulate over the years, with a surge in the past five years, more and more scientists, doctors, and organizations are acknowledging the importance of a field that some are calling environmental cardiology.

One group that is beginning to embrace environmental cardiology is the American Heart Association (AHA), an 80-year-old organization that has traditionally focused on risk factors such as poor diet and lack of exercise as some of the most important contributors to CVD. In the 1 June 2004 issue of *Circulation*, an expert panel of 11 researchers and physicians published an AHA Scientific Statement that concluded that air pollutants, one of the major environmental exposure sources under investigation by environmental cardiologists, pose a “serious public health problem” for CVD. This is the first official AHA acknowledgment of such links.

The group’s decision was based on the breadth and depth of the accumulating information. “There was no single major study that prompted the writing of this paper,” says Sidney Smith, past president of the AHA and a professor of medicine at the University of North Carolina at Chapel Hill. “It was the gathering body of evidence that connected air pollution with cardiovascular diseases, extending well beyond cigarette smoke.”

The AHA paper was a very positive development in the eyes of some of the researchers who have been involved in the field for many years. “That’s pretty amazing,” says C. Arden Pope III, an environmental epidemiologist at Brigham Young University. “It’s taken the research out of the fringes and made it part of the mainstream.”

Less than two months after the release of the AHA statement, the U.S. Environmental Protection Agency (EPA) gave a clue to how seriously it takes this issue, awarding the largest scientific research grant in its history, $30 million, to study links between air pollution and CVD. The research team will be headed by associate environmental and occupational health professor Joel Kaufman of the University of Washington, and includes scientists from nine other universities and medical centers.

A few other government agencies, such as the National Heart, Lung, and Blood Institute (NHLBI), have also begun to address the links between environmental agents and CVD, as have advocacy organizations such as the American Lung Association and the Natural Resources Defense Council. And the NIEHS, one of the original players in the environmental cardiology arena, has ramped up its efforts to explore this area of research.

There is still a ways to go before environmental cardiology is fully embraced as a medical paradigm. Many major public health organizations, such as the World Health Organization (WHO) and the Centers for Disease Control and Prevention (CDC), have yet to fold this concept into their prevention efforts in any significant way. And there is very little trickle-down into the typical doctor–patient relationship.

Nonetheless, environmental cardiology shows signs of increasingly becoming a factor in research, public policy discussion, and pollutant regulation, as its presence spreads into journals, conferences, textbooks, e-mail discussion groups, and continuing medical education courses. Even The Weather Channel is getting into the act with a new feature that advises viewers on daily levels of pollutants that can affect heart health.

## A Heavy Burden for Hearts Worldwide

The most basic facts about CVD haven’t been available for very long. The early decades of the 1900s, when physicians were just beginning to form groups to address heart diseases, were a time of “almost unbelievable ignorance” about these conditions, according to the AHA website.

That has changed, spurred in large part by the huge impact CVD has on people. Heart conditions such as heart attack and congestive heart failure are the leading killer in the United States, and stroke is third, according to the CDC’s *Deaths: Preliminary Data for 2002*, released in February 2004. Combined, these two categories of CVD alone account for about 35% of all U.S. deaths, compared to 23% for cancers. Other serious health problems that fall into the CVD classification include aortic aneurysms, high blood pressure, and congenital cardiovascular defects.

CVD deaths had been declining sharply in the United States over the past few decades, but that curve has flattened out in recent years. Death rates for heart diseases (responsible for nearly 696,000 U.S. deaths in 2002) declined about 3% from 2001 to 2002, as did death rates for stroke (responsible for about 163,000 U.S. deaths in 2002). But death rates attributed to high blood pressure (responsible for about 20,000 U.S. deaths in 2002) rose about 3%, continuing a steady rise over the prior 20 years.

Other industrialized nations have seen similar patterns. The WHO says that CVD accounts for about one-third of global deaths, killing about 16.7 million people each year. Patterns in developing countries are quickly emulating those in developed countries, thanks to the imported western lifestyle and reductions in infectious disease deaths and other acute causes of death. The WHO estimates that CVD will be the leading killer in developing countries by 2010.

But there are huge variations from country to country. In 36 countries tracked by the AHA, CVD death rates differ dramatically, with rates in some of the most-affected countries, such as the Russian Federation, Bulgaria, and Romania, more than five times higher than in some of the least-affected countries, such as France, Japan, and Australia. Variations in factors such as diet, exercise, smoking, health care quality and availability, and pollution likely play a role in these differences.

Within a country, there also can be huge variations. In the United States, the CVD rate in the least-affected state, Minnesota, is less than 60% the rate in the most-affected state, Mississippi, according to the AHA’s *Heart Disease and Stroke Statistics—2004 Update*. And the gap has been widening. Minnesota had a 27% decline from 1990 to 2000, while Mississippi saw a 12% decline.

Race and ethnicity are significant risk determinants. Black women in the United States are 2.5 times as likely as Asian and Pacific Islander women to die of diseases of the heart, and a similar ratio of 2.25 to 1 holds for black men, according to CDC statistics published in *Women and Heart Disease* and *Men and Heart Disease*. Death rates for American Indian, Alaska Native, Hispanic, and white men and women fall in between these two extremes.

Similar disparities exist for stroke, with the death rate for black men and women more than twice that of the least-affected groups—Hispanics, American Indians, and Alaska Natives—according to the CDC’s 2003 publication *Atlas of Stroke Mortality: Racial, Ethnic, and Geographic Disparities in the United States*.

The elderly tend to be most vulnerable to CVD, and the problem is expected to worsen in many countries as populations age. But sudden cardiac-related deaths have increased dramatically among people under age 35, according to the CDC’s 2003 report *A Public Health Action Plan to Prevent Heart Disease and Stroke*. And CVD is the third-leading cause of death for children under 15, according to the AHA.

Deaths aren’t the only consideration. Chronic diseases, which the CDC says affect more than 90 million people in the United States alone, are often due to CVD. Diseases of the heart, high blood pressure, and stroke underlie about one of every five U.S. chronic disease cases.

CVD also includes cardiac birth defects. Among structural birth defects, cardiovascular malformations are the most common among live births, affecting 1 baby in 125, according to the March of Dimes. They are also the leading cause of birth defect–related infant deaths.

There is also growing evidence that prenatal exposures to some environmental pollutants, such as solvents, pesticides, and dioxins, may result in subtle functional abnormalities that show up as disease in adulthood. In the area of CVD, this hypothesis—although important—is only just beginning to receive much attention.

## The Role of the Environment: A Change of Heart

Until the past few years, the huge worldwide toll of CVD had been attributed primarily to lifestyle factors such as poor diet, lack of exercise, lack of medical care, smoking, and exposure to secondhand smoke. A large worldwide study published 11 September 2004 in *The Lancet* found that nine lifestyle and biological factors accounted for 90% of the risk for one major form of CVD, heart attack, with two factors—cigarette smoking and an abnormal ratio of blood lipids—accounting for two-thirds of that risk.

But public health officials still haven’t been able to explain the cause behind a significant number of CVD deaths, says Aruni Bhatnagar, a professor of medicine and project leader of the University of Louisville’s Center of Environmental Cardiology, in a February 2004 article in the *American Journal of Physiology—Heart and Circulatory Physiology*.

Clues about environmental influences began to show up decades ago. For instance, beer containing elevated concentrations of cobalt (used for quite a few years around the world to retain the beer’s head) were found in the 1960s to contribute to cardiomyopathy in some drinkers. Studies by the National Toxicology Program have examined links between numerous environmental agents and cardiovascular effects for several decades, says NIEHS chemist June Dunnick. And a 1985 study of chain saw exhaust published in the *European Journal of Respiratory Diseases* found an increase in carboxyhemoglobin, which makes it harder for the blood to carry oxygen and can lead to serious nerve damage.

By 1994, a number of published studies had found links between smoking and CVD. Evidence against smoking and secondhand smoke continues to grow, and among the chemical exposures with potential CVD links, tobacco smoke is the most worrisome so far, says William Farland, acting deputy assistant administrator for science in the EPA Office of Research and Development.

A few researchers saw some of these early clues about various chemicals and shifted their work into the environmental realm. Eliseo Guallar, an assistant professor of epidemiology at The Johns Hopkins University, studied nutritional aspects of fish oils for several years, but saw no conclusive evidence of benefits. Then he saw a study on mercury in fish, and the light went on. “For a long time, it never occurred to me that, when you eat fish, you eat contaminants,” he said. “I read this and I said, ‘Of course!’” He shifted his research, and published a study in the 28 November 2002 *New England Journal of Medicine* that found that mercury canceled out the benefits of fish oils and contributed to an increased risk of heart attack.

Others were following a parallel path. In 2000, University of Louisville researchers coined the term “environmental cardiology,” Bhatnagar says, and established the Center of Environmental Cardiology in the university’s Health Sciences Center. Wayne Cascio, chief of the Division of Cardiology at East Carolina University’s Brody School of Medicine, says he came up with the same term of “environmental cardiology” on his own, and Harvard’s John Godleski, an associate professor of environmental health who has published eight related papers in recent years, uses the term as well.

Others, such as Farland, have heard the term and think it’s appropriate, especially for bringing cardiologists into the field, but aren’t using it routinely. Thomas Burke, a professor of health policy and management at the Johns Hopkins Bloomberg School of Public Health, uses a long version, “environmental influences on cardiovascular health,” but likes “environmental cardiology.” Guallar thinks the term “cardiology” is a little too clinically oriented, and uses the term “cardiovascular disease epidemiology.”

Whatever term is used, by 2002 the notion of environmental cardiology was no longer considered outlandish after other epidemiological studies, and some studies of biological processes, found numerous links between different chemicals and CVD. That year, the NIEHS, the EPA, NHLBI, the AHA, and St. Jude Medical convened a workshop in Durham, North Carolina, and scores of participants discussed the links between environmental agents and CVD. Environmental cardiology even began to creep into the public consciousness with the Food and Drug Administration’s 2004 ban of dietary supplements containing ephedrine alkaloids, based on evidence that the substances are linked to heart attack and stroke.

## Fingering the Villains

Given the evidence so far, fine particulates appear to be one of the primary environmental villains linked with CVD, and are drawing much of the research interest. One of the first major developments was publication of findings from the Six Cities Study in the 9 December 1993 *New England Journal of Medicine*. The study, by Harvard environmental epidemiologist Douglas Dockery, Pope, and six others, found a significant link between ambient urban air pollution, especially fine particulates, and increased deaths caused by cardiopulmonary disease, along with increases in lung cancer. In January 2004, *Circulation* published a study by Pope and others covering more than half a million people over 16 years, which found that fine particulates were more strongly linked with deaths from cardiovascular causes than with deaths from respiratory causes. The pattern of cardiovascular deaths was consistent with evidence that mechanistic pathways linking exposure and death included pulmonary and systemic inflammation, accelerated atherosclerosis (hardening of the arteries), and changes in cardiac autonomic function (as measured by changes in heart rate variability).

In addition to particulates, dozens of other substances have been identified as playing some role in CVD. The other five EPA “criteria” air pollutants (ozone, carbon monoxide, nitrogen oxides, sulfur dioxide, and lead) have some evidence linking them with CVD, as do at least 17 of the 87 drinking water contaminants monitored by the EPA. At least 8 of the 116 contaminants in people tracked so far in the CDC’s ongoing biomonitoring project have CVD links. Pat Mastin, chief of the NIEHS Cellular, Organ, and Systems Pathobiology Branch, points out that several occupational exposures also have been associated with CVD, including exposures to vinyl chloride (used to produce polyvinyl chloride and industrial solvents), carbon monoxide (a common exhaust gas), and allylamine (used in ion exchange resins, pharmaceuticals, and water-soluble polymers).

According to Mastin, arsenic has been linked in Asia with a condition known as black foot disease, so named because of the gangrene caused by severe disease in the blood vessels. In addition, he says, areas of the United States have high drinking water concentrations of arsenic, and there is evidence for a link between arsenic exposure and ischemic heart disease and hypertension in such areas. And there are more than 50 other substances, including many heavy metals, solvents, and a few pesticides, that have been implicated in CVD by other sources.

The research conducted so far has found many tangible indicators of cardiovascular system effects, including atherosclerosis; vasoconstriction; and changes in heart rate variability, blood pressure, coagulation, platelet activation, endothelial cells, and the clotting protein fibrinogen. Those changes have been linked with serious outcomes such as ischemic heart disease, congestive heart failure, acute myocardial infarction, malignant ventricular arrhythmias, plaque vulnerability, acute thrombosis, stroke, and hypertension.

The difficult job of figuring out exactly how various chemicals cause these problems has just begun. Many pathways are under investigation. On the top of the list for some is the systemic inflammation caused by some chemicals. “In the end, it’s all going to be tied to inflammation,” Cascio says.

Pope, who agrees inflammation is a key element, has another prime suspect. “There’s simply a lot of evidence that there’s a role for [effects on] autonomic function,” he says.

Cellular changes, such as alterations in ion channel function, cell proliferation, signal transduction pathways, and cell signaling, also are under scrutiny. For instance, research by Armando Meyer and colleagues published in the February 2004 *EHP* found that the insecticide chlorpyrifos affects cell signaling cascades critical to cardiac homeostasis.

Problems may be caused not just by a chemical, but by its metabolites. In a study reported in the September 2004 issue of *Toxicological Sciences*, Dunnick and NIEHS colleague Abraham Nyska found that bis(2-chloroethoxy)methane caused mitochondrial damage in hearts in a rodent model system. They hypothesized that the thiodiglycolic acid, a metabolite of bis(2-chloroethoxy)methane as well as many other chemicals, causes this chemical-related mitochondrial damage and heart toxicity. Dunnick and Nyska observed a biphasic response: initial damage to myocytes was repaired in an apparent temporary adaptive response that the animals were no longer able to launch as they aged.

Other areas of concern include genetic variations and expression, gene polymorphisms, oxidative stress, protein expression, and post-translational modifications. In addition, many researchers suspect indirect links with the immune, pulmonary, and neurological systems.

## Seeking the Heart of the Matter

Millions of dollars are being pumped into environmental cardiology research. The largest single award is the $30 million granted in July 2004 to the University of Washington–led team, which will focus on the effects of fine particulates. The 10-year study will evaluate about 8,700 people in six states, representing a variety of ethnic groups, for clinical and subclinical effects such as heart attack, stroke, and atherosclerosis.

The University of Louisville Center of Environmental Cardiology was awarded a five-year $7 million NIEHS grant in 2003 for studies on the effects of aldehydes, which are found throughout the environment, making up a large part of typical urban air pollution and also showing up in food and drinking water. The NIEHS also awarded $3 million in 2003 to environmental epidemiologist Ralph Delfino at the University of California, Irvine, and his colleagues to study the effects of fine particulates in the elderly. The study, expected to conclude in 2007, will investigate a range of CVD effects, in part through intensive personal monitoring, and will address seasonal and geographic variations. The EPA and the NIEHS awarded a dozen grants in September 2004, totaling about $4 million, that focus on a variety of links between particulates and CVD. The topics range from acute effects of particulates on the autonomic nervous system to chronic effects of particulates on atherosclerosis, Mastin says.

The Health Effects Institute, funded by the EPA and industry, is backing several related studies, says institute senior scientist Geoffrey Sunshine. A University of Rochester study looking at the effects of particulates on CVD is expected to be published by November 2004. Dockery is looking at whether particulate exposures may make internal cardiac defibrillators fire more frequently. And Annette Peters, head of the Institute of Epidemiology at Germany’s government-funded GSF–National Research Center for Environment and Health, and her colleagues are investigating the effects of fine particulates on nonfatal myocardial infarction. Many other related studies are being conducted around the world.

Karen Kuehl, a professor of pediatrics and a cardiologist at Children’s National Medical Center in Washington, D.C., has participated in a number of studies of environmental influences on cardiovascular birth defects, but laments the limited funding available, possibly due to the minor influence infants have on the budget process. “Babies don’t vote,” she says with a short laugh. Nonetheless, the NIEHS has begun to make this area more of a priority, and issued the program announcement “Environmentally Induced Cardiovascular Malformations” in 2002. Mastin, whose branch administers this program, says research funded under these grants is examining how prenatal exposures influence the risk of cardiovascular birth defects.

Related information may also eventually come out of the chronic disease tracking efforts of the CDC’s Environmental Public Health Tracking Program, says Burke, who heads one arm of the CDC effort. The fledgling program, begun in 2002, is designed to eventually provide extensive data documenting links between the presence of environmental agents, exposures, and ensuing diseases, including CVD.

From a policy perspective, the AHA is continuing to develop official policy on the links between pollutants and CVD. It’s also advocating and supporting further research to help it determine whether pollutants will rise to the level of an actual risk factor in its perception, meaning that it is a significant, independent contributing factor to CVD. The evidence isn’t quite there yet, according to Smith.

Although the links between chemical exposures and CVD are becoming more widely recognized, few public health agencies have responded yet. The WHO is focusing most of its efforts on preventing CVD caused by factors such as diet, exercise, and smoking, which it says account for 75% of CVD, though it acknowledges that pollutants other than cigarette smoke are a cause for concern. In the United States, the CDC updated its *Public Health Action Plan to Prevent Heart Disease and Stroke* in 2003, but the plan still pays scant attention to environmental contaminants, though it acknowledges pollutants are an issue. NHLBI, too, acknowledges the issue of chemical exposures, says George Sopko, a cardiologist at that institute, but has not given it much weight yet. Few, if any, state health programs address environmental cardiology in any significant way, according to several state health officials.

## Riding the Learning Curve

The venues for professionals interested in learning more about the links between environmental agents and CVD are expanding. One journal that focuses extensively on related issues of cardiovascular toxicities of drugs, novel therapies, and environmental pollutants is *Cardiovascular Toxicology*, which began publishing in 2001. Among the general journals that have published related studies are *EHP*, *JAMA*, the *New England Journal of Medicine*, *Circulation*, *Inhalation Toxicology*, *Toxicologic Sciences*, *Epidemiology*, and the *American Journal of Epidemiology*.

The 2004 reference book *Netter’s Cardiology* has a chapter on the topic, authored by Cascio, and the issue is increasingly appearing on the agenda of conferences run by organizations such as the AHA, the American Thoracic Society, the International Society of Environmental Epidemiology, the NIEHS, and the EPA. Discussions on the latest developments routinely occur on the NIH e-mail group EnviroHeart (**http://list.nih.gov/archives/enviroheart.html**).

Interested doctors may soon be able to learn more through continuing medical education programs. The EPA is working on a certification program to educate doctors about ozone and respiratory effects, says agency environmental health scientist Susan Stone, and the agency anticipates following that up with a program on particulates that will cover cardiovascular effects.

At the moment, though, doctors have no accepted medical treatments that are known to be effective in reducing the effects of chemicals on CVD, says Robert D. Brook, who is lead author of the AHA Scientific Statement, an assistant professor of medicine at the University of Michigan, and a practicing physician. Instead, concerned people should avoid exposures to the extent possible, he says.

One tool to help people avoid air pollution exposures is the EPA’s AIRNow website (**http://www.epa.gov/airnow/**), Stone says. Daily reports on local particulate and ozone levels can help people decide whether to limit activity and thus, exposure. That idea will be incorporated into a Weather Channel feature called “Air Aware” that the EPA is collaborating on, which is scheduled for launch in mid-autumn 2004.

The EPA also is finalizing an educational poster designed to be hung in doctors’ offices and elsewhere, which is expected to be released in November 2004. It devotes about half its space to the effects of a few common pollutants on the cardiovascular system. The other half addresses respiratory effects. In 2003 the agency released an educational brochure on particulates that folded in some information on cardiovascular effects. The brochure has been distributed by some state and local air agencies, and is available to the public through the EPA’s National Center for Environmental Publications.

## Reaching a Regulatory Threshold

Given the relatively early stages of wide-scale research into the links between chemicals and CVD, it likely will be some time before any regulations change. “I wish new science were incorporated more rapidly, but I’m not kidding myself that the EPA is going to hop to it on this one,” says Gina Solomon, a senior scientist with the Natural Resources Defense Council and a practicing physician.

Greg Dana, vice president of environmental affairs with the Alliance of Automobile Manufacturers, hopes she is correct. He says that vehicle pollution, which accounts for a large part of the environmental load of particulates and other air pollutants, has already been addressed enough. “There’s a pretty big onslaught of rules and regulations in coming years that will take care of a lot of emissions,” he says. “Hopefully we’ve addressed whatever concerns are being raised.”

Dana points to a number of emissions regulations set to phase in over the next several years. Federal “Tier 2” regulations set by the Clean Air Act Amendments of 1990 will phase in between 2004 and 2009, and will reduce car and light truck emissions by 80% over today’s cars. Beginning in model year 2007, Tier 2 regulations will reduce heavy-duty vehicle nitrogen oxide emissions by 90% and particulate emissions by 95%. Both of these rules also contain provisions to remove sulfur from gasoline and diesel fuel. In addition, “maximum achievable control technology” standards are now final for 110 industry categories covering almost all business sectors in the country to control air toxics. “These are three of the biggest [rules], but there are others that will reduce emissions even more,” Dana says.

If additional regulations are adopted, they might address concentrations of allowable exposure, time period of exposure, and vulnerable populations, says Farland. He notes the EPA is already incorporating some CVD concerns into discussions about the next-generation fine particulate standard under consideration (which will be published as a proposal by 31 March 2005 and finalized by 20 December 2005). Guallar also notes that environmental cardiology research might shift cost–benefit calculations used in assessing new regulations.

Although the regulatory outlook is unpredictable, and much of the science and medicine is in its early stages, the rapidly expanding evidence appears to be carrying the newly recognized field of environmental cardiology into ever-widening areas of influence. Says Pope, “It’s remarkable what’s happened in the last five to six years. I think we’re making a lot of progress.”

## Figures and Tables

**Figure:**
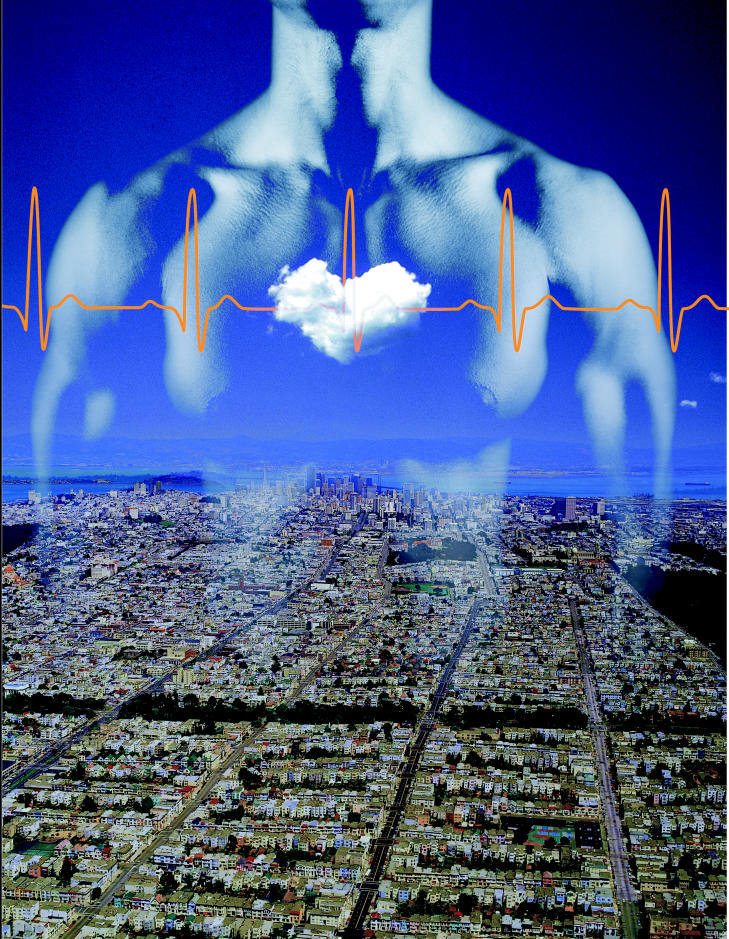


**Figure:**
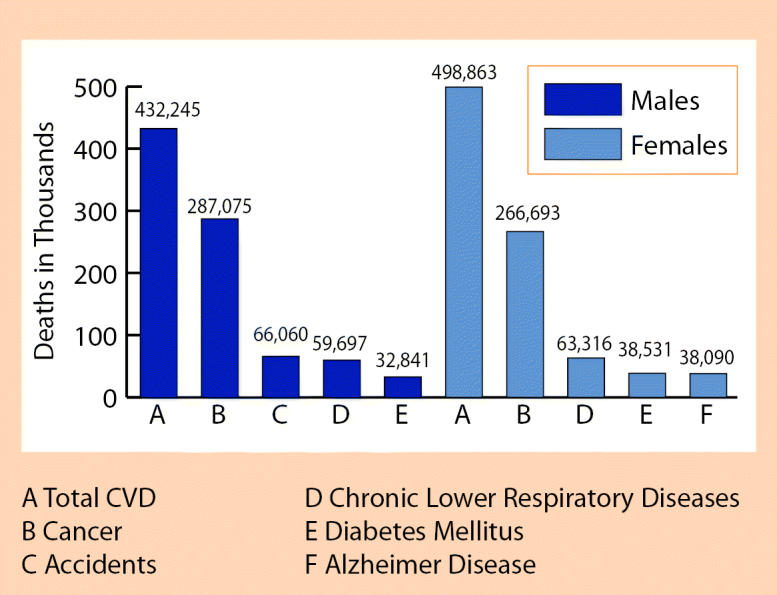
**Leading Causes of Death for All Males and Females** United States, 2001

**Figure:**
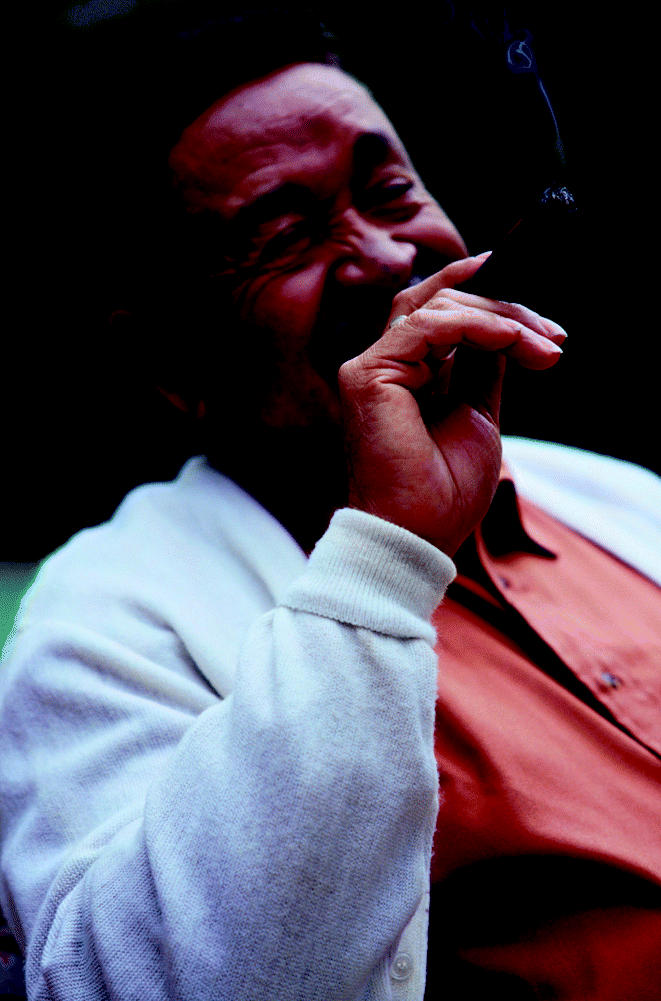
**Smoking gun.** In study after study, tobacco smoke is one of the pollutants most directly linked with CVD.

**Figure:**
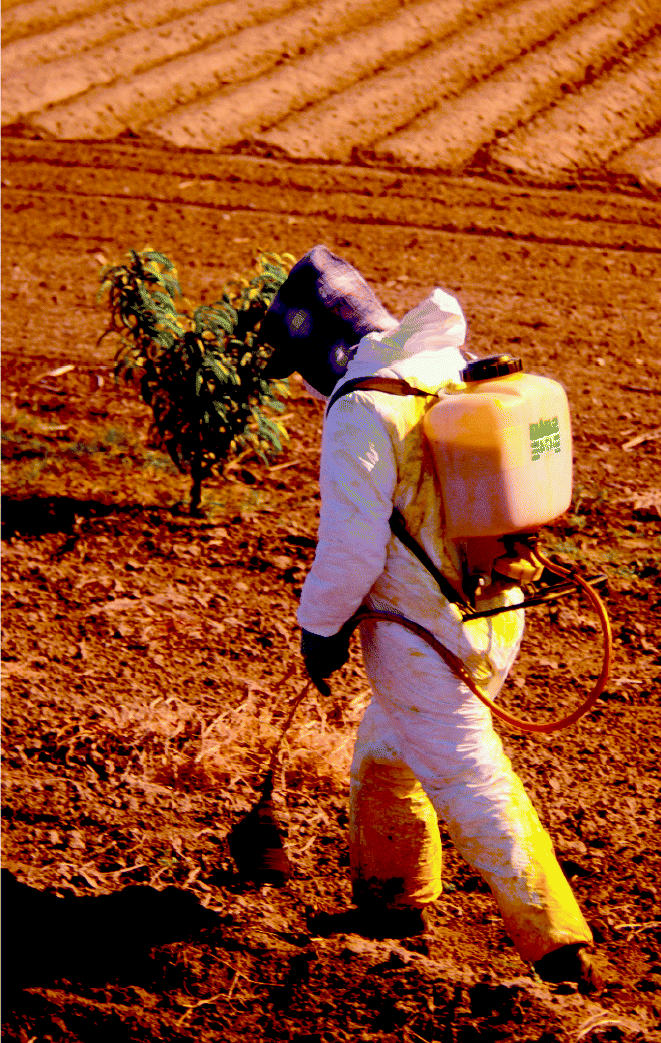
**Chemicals and cardiology.** Research studies have linked certain pesticides with CVD, including some effects in children.

**Figure:**
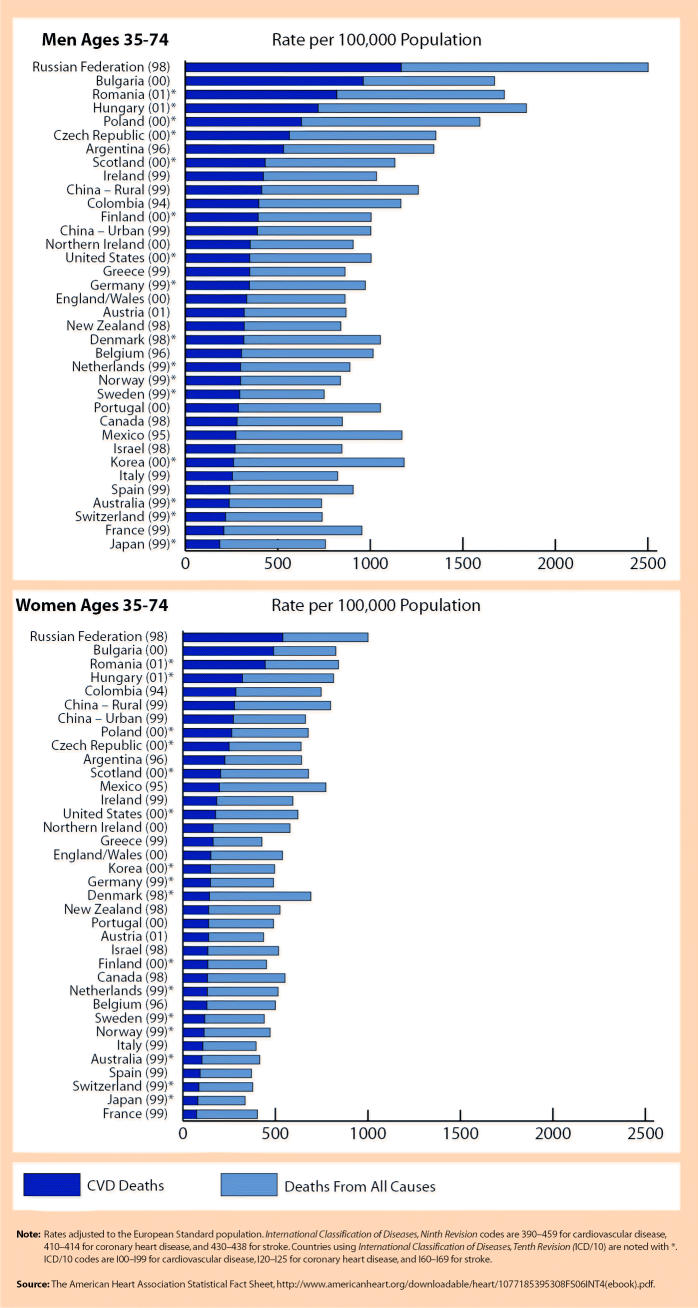
Death Rates for Total Cardiovascular Disease, Coronary Heart Disease, Stroke, and Total Deaths in Selected Countries (most recent year available)

**Figure:**
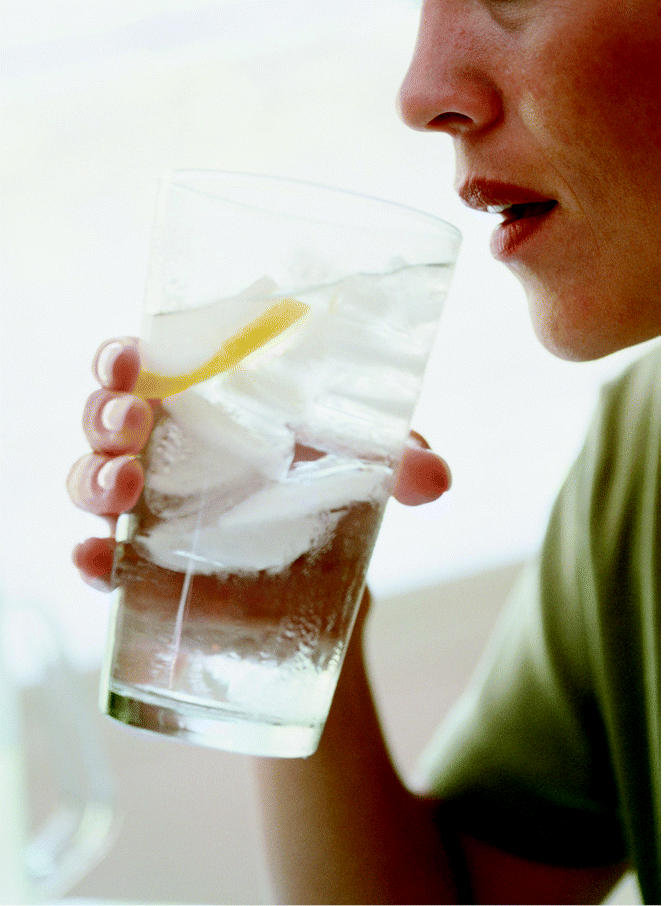
**A diet for disease?** New research will look at links between CVD and chemical contaminants in food and water.

**Figure:**
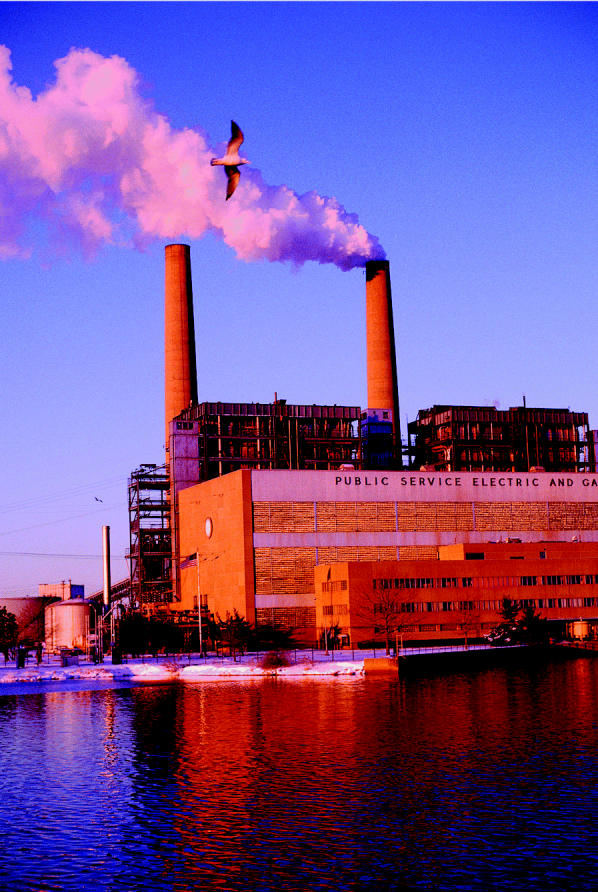
**Particularly offensive.** Fine particulate air pollution is being investigated as a contributor to effects such as heart attack, stroke, and atherosclerosis, especially in the elderly.

**Figure:**
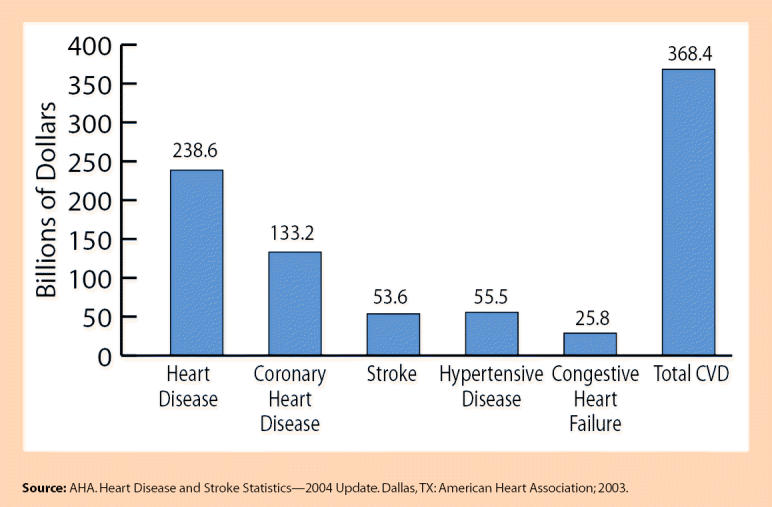
Estimated Direct and Indirect Costs of Cardiovascular Disease and Stroke

**Table:** **Children,Youth, and Cardiovascular Disease**

				**Non-Hispanic Whites**		**Non-Hispanic Blacks**		**Mexican Americans**	
**Diseases and Risk Factors**	**Total Population**	**Total Males**	**Total Females**	**Males**	**Females**	**Males**	**Females**	**Males**	**Females**
Congenital Defects									
Mortality 2001 (all ages)	4.1 K	2.1 K	1.9 K	1.8 K	1.5 K	0.4 K	0.3 K	—	—
Mortality 2001 (< age 15)	2.1 K	1.1 K	1.0 K	—	—	—	—	—	—
Tobacco									
Prevalence grades 9–12:									
Current tobacco use 2001	—	38.5%	29.5%	—	—	—	—	—	—
Current cigar use 2001	—	22.1%	8.5%	—	—	—	—	—	—
Smokeless tobacco use 2001	—	14.8%	1.9%	—	—	—	—	—	—
High school students:									
Used tobacco in last 30 days	—	—	43.4%	32.3%	21.6%	17.4%	31.5%	27.2%	
Blood Cholesterol									
Ages 4–19:									
Mean total cholesterol mg/dL	165	—	—	162	166	168	171	163	165
Ages 4–19:									
Mean HDL cholesterol mg/dL	—	—	—	48	50	55	56	51	52
Ages 12–19:									
Mean LDL cholesterol mg/dL	—	—	—	91	100	99	102	93	92
Physical Inactivity									
Prevalence 2001 grades 9–12:									
Vigorous activity last 7 days	—	—	—	73.7%	59.8%	72.4%	47.8%	68.8%	52.4%
Moderate activity last 7 days	—	—	—	29.8%	24.7%	23.7%	16.5%	25.9%	18.5%
Overweight									
Prevalence 2001:									
Preschool children ages 2–5	>10%	—	—	10%		8%		11%	
Children ages 6–11	3.8 M (15.3%)	2.0 M (16.0%)	1.8 M (14.5%)	11.9%	12.0%	17.6%	22.1%	27.3%	19.6%
Adolescents ages 12–19	5.0 M (15.5%)	2.6 M (15.5%)	2.4 M (15.5%)	13.0%	12.2%	20.5%	25.7%	27.5%	19.4%
Students grades 9–12	—	—	—	12.4%	5.3%	17.5%	14.6%	21.3%	8.8%

**Note:** K = thousands; M = millions; mg/dL = milligrams per deciliter; (−) = data not available. Overweight in children is body mass index (BMI) at 95th percentile of the Centers for Disease Control and Prevention 2000 growth chart. Death rates are age-adjusted per 100,000 population, based on the 2000 U.S. standard.

**Source:** AHA. Heart Disease and Stroke Statistics—2004 Update. Dallas,TX: American Heart Association; 2003.

